# Media Exposure, Behavioural Risk Factors and HIV Testing among Women of Reproductive Age in Papua New Guinea: A Cross-Sectional Study

**DOI:** 10.3390/tropicalmed7020030

**Published:** 2022-02-18

**Authors:** Oyelola A. Adegboye, Henry C. Ezechukwu, Hannah Woodall, Megan Brough, Jodie Robertson-Smith, Rosella Paba, Geraint Czech, Theophilus I. Emeto

**Affiliations:** 1 Public Health and Tropical Medicine, College of Public Health, Medical and Veterinary Sciences, James Cook University, Townsville, QLD 4811, Australia; oyelola.adegboye@jcu.edu.au (O.A.A.); hannah.woodall@my.jcu.edu.au (H.W.); megan.brough@my.jcu.edu.au (M.B.); jodie.robertsonsmith@my.jcu.edu.au (J.R.-S.); 2 World Health Organization Collaborating Center for Vector-Borne and Neglected Tropical Diseases, College of Public Health, Medical and Veterinary Sciences, James Cook University, Townsville, QLD 4811, Australia; 3 Australian Institute of Tropical Health and Medicine, James Cook University, Townsville, QLD 4811, Australia; 4 Department of Medical Biochemistry, EKO University of Medicine and Health Sciences, Ijanikin, Lagos 102004, Nigeria; hezechukwu@ekounimed.edu.ng; 5 Rural Medical Education Australia, Griffith University, Griffith University Parklands Drive, Southport, QLD 4222, Australia; 6 College of Medicine and Dentistry, James Cook University, Townsville, QLD 4811, Australia; rossella.paba@my.jcu.edu.au; 7 History, Cultural Heritage and Territory, University of Cagliari, Piazza Arsenale 1, 09124 Cagliari, Italy; 8 Emergency Department, Royal Darwin Hospital, 105 Rocklands Drive, Tiwi, NT 0810, Australia; czechy@icloud.com

**Keywords:** HIV, mass media, Papua New Guinea, knowledge, awareness

## Abstract

Background: Reproductive health remains a major health concern in developing countries such as Papua New Guinea (PNG). The prevalence of human immunodeficiency virus (HIV) in PNG is the highest in the Southern Pacific region, with women having a higher risk of contracting the infection. Hence, there have been several policies aimed at mitigating the spread of the disease. One of these policies include the use of mass media as a health promotion tool to educate the population on the risk of the disease. Therefore, this study aimed at investigating the association of mass media to HIV testing among women. Methods: Data were obtained from the PNG Demographic and Health Survey (DHS) of 2019. A total of 15,005 reproductive-age women was included in this analysis. Results: The results showed that women with low (aOR = 1.63, 95% CI: 1.39, 1.90) and high (aOR = 1.53, 95% CI: 1.36, 1.72) media exposure were more likely to undertake HIV testing compared to those with no media exposure. Compared to no education, women with incomplete primary (aOR = 1.22, 95% CI: 1.06, 1.40), complete primary (aOR = 1.56, 95% CI: 1.30, 1.87), incomplete secondary (aOR = 2.18, 95% CI: 1.85, 2.58), complete secondary (aOR= 2.33, 95% CI: 1.77, 3.09) and higher (aOR = 3.38, 95% CI: 2.57, 4.46) education were more likely to undertake HIV testing. Compared to women with the poorest wealth index, women with richer indexes were more likely to undertake HIV testing. Women living in rural areas were less likely to undertake HIV testing (aOR = 0.72, 95% CI: 0.63, 0.82). However, marital status, knowledge of transmission and religion were not associated with HIV testing. Conclusion: In conclusion, this study provides strong evidence that mass media exposure increases the likelihood of HIV testing in women of reproductive age in PNG. Mass media campaigns would serve as a cost-effective health promotion tool against the spread of disease.

## 1. Introduction

The prevalence of human immunodeficiency virus/acquired immune deficiency syndromes (HIV/AIDS) in the Southern Pacific region is reported to be 0.2% [1]. However, the estimated prevalence in Papua New Guinea (PNG) is 0.9, the highest in the region [2]. PNG is a culturally diverse and geographically varied Pacific Island nation, and HIV prevalence varies geographically within the country [3]. Focal epidemics exist amongst higher risk groups, as reported in the 2018 *Kauntim Mi Tu*, a multi-site summary report with key findings of HIV burden amongst female sex workers (15.5%) and men who have sex with men and transgender women (7.7%) [3,4].

Overall, women are at a higher risk of transmission than men, i.e., 1.1% vs. 0.7% [2]. The role of women in controlling the spread of HIV is also of particular interest due to vertical (mother to child) transmission [5,6] and the positive correlation between gender-based violence and increased risk of HIV transmission, particularly considering the high prevalence of violence against women and children in PNG [4,7,8,9,10]. The UNAIDS 90-90-90 targets stated that by 2020, 90% of people living with HIV would be aware of their status, 90% of those diagnosed with HIV would be on appropriate antiretroviral treatment, and 90% of those on treatment would demonstrate viral suppression [11]. The role of women in PNG society strongly impacts their health status and ability to access healthcare [4,12]. Although it was estimated that 71.0% of Papua New Guineans living with HIV are aware of their status, the frequency of pregnant women undertaking HIV testing in 2019 was low, at 19.5%, despite a vertical transmission rate of 22.6% [2]. Similarly, only 39.6% of TB cases were tested for HIV in 2015, even though TB is a common HIV comorbidity [13]

In a resource-limited country such as PNG, it is crucial to identify ways to improve risk-taking behaviours and uptake of HIV testing to enable effective planning and policies. In response to the HIV epidemic, the PNG government introduced a Nationwide HIV/AIDS Support Program between 2000 and 2005 and another National HIV Prevention Strategy scheme between 2010 and 2015 with the initiative of rapid scaling up HIV counselling, testing, surveillance and anti-viral therapy [14]. Despite this, other looming risk factors such as religion barriers, myths, stigma, cultural barriers have negatively influenced the governmental approach [9,15,16]. 

Knowledge about HIV among young people is low; a deeper understanding of HIV/AIDS transmission would be an effective tool in practising the preventive measures, which are not limited to condom use and HIV testing [17]. HIV testing among the younger population and women is crucial, and this could serve as a preventive intervention in reducing the incidence of HIV/AIDS in the community.

Mass media are a common and crucial health education instrument that is employed to disperse health information and health-promotion activities to a large proportion of the population at a relatively low cost [14] Previous studies have shown that media can positively influence HIV testing rate [18,19] and other vital areas of public health issues [20]. An earlier impact data study by Turk and colleagues in 2017 indicates that the media can positively influence HIV test rates as well as unhelpful/stigmatising attitudes and behaviours [14].

A review of the literature revealed a paucity of contemporary data regarding the relationship between media exposure and HIV testing and HIV risk-taking behaviours in PNG. Thus, this paper examines the impact of media exposure and other behavioural risk factors on HIV testing amongst women of reproductive age in Papua New Guinea. Understanding the association between mass media campaigns and the characteristics of the population and HIV testing rates can assist in directing future campaigns, the allocation of resources, and the effectiveness of such campaigns.

## 2. Materials and Methods

### 2.1. Study Area and Data Sources

**Figure 1:**
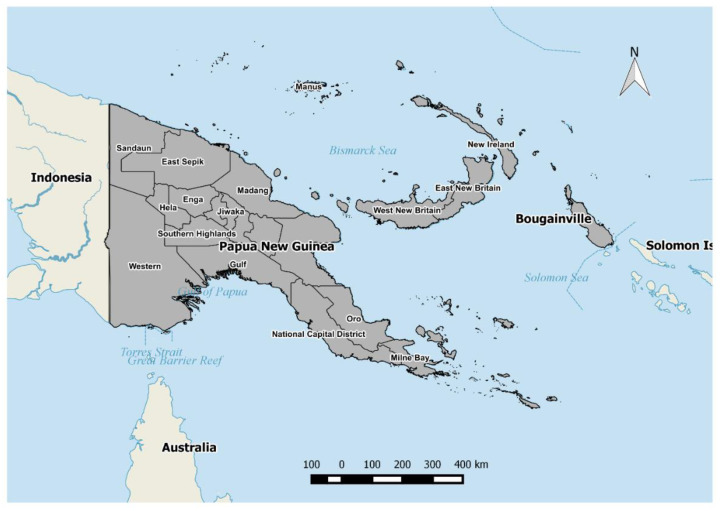
Map showing the study region, PNG.

The study was carried out in PNG, which lies north of Australia (Figure 1). The estimated population of PNG is approximately 9 million, with greater than 80% of this population based in rural or remote areas [12]. The study was based on the nationally representative 2016–2018 PNG Demographic and Health Survey (NDHS) Data [21]. The 2016–2018 PNG DHS used a stratified two-stage cluster sampling design stratified by location—rural and urban. The PNG DHS team ensured that urban and rural regions were sampled with probability proportional to segment size. Details of the sample design can be found in the PNG DHS report [21].

The PNG DHS survey contained information on the following: family planning, maternal and child health and nutrition, childhood mortality, malaria, female genital cutting, sexual activity, marriage, HIV/AIDS and sexually transmitted diseases/infections [21]. For this study, we considered the data of women aged 15–49 years.

### 2.2. Measures

#### 2.2.1. Outcome Variable

The primary outcome variable for this study was HIV testing (dichotomised as Yes or No).

#### 2.2.2. Covariates

##### Media Exposure

The variables “frequency of reading print media”, “frequency of listening to radio” and “frequency of watching television” were used to calculate the “media exposure score”. Each variable was coded as 0 = *not at all*, 1 = *less than once a week*, 2 = *at least once a week*; the computed “media exposure” index ranged from 0 to 6 and was categorised as “No exposure: 0”, “low exposure: 1–3” and “high exposure: ≥4”.

##### Knowledge of HIV/AIDS Transmission

The following questions were asked: “Can one get HIV from mosquito bites”, “Can one get HIV by sharing food with a person who has AIDS”, “Can one get HIV by witchcraft or supernatural means”, “Ever heard of a Sexually Transmitted Infection (STI)”, “Ever heard of AIDS”, “Reduce risk of getting HIV: always use condoms during sex”, “Reduce risk of getting HIV: have one sex partner only, who has no other partners” and “Can a healthy-looking person have HIV”. Each was recoded to create an “HIV/AIDS transmission knowledge” score, ranging from 8 (good knowledge of HIV/AIDS transmission) to 0 (poor knowledge of HIV/AIDS transmission). 

##### Other Covariates

Other variables include behavioural variables such as condom use, socio-demographic variables such as age at first sex, age at first cohabitation, current age, education attainments, wealth index and religion. See Table 1 for the full list of variables considered in this study.

### 2.3. Data Analysis

In this study, categorical variables are presented as frequencies and percentages, while medians and interquartile ranges (IQR) as continuous variables. Data were analysed using the chi-square test or Mann–Whitney U test to examine the difference in HIV testing and media exposure among variables. Univariate and multivariable binary logistic regression was used to examine the effect of covariates on the uptake of HIV testing. The results are presented as odds ratio (OR) and adjusted odds ratio (aOR), together with their 95% confidence interval. The level of statistical significance was set at a 5% alpha level. Data were analysed in R Version 4.0.2.

## 3. Results

The characteristics of the study participants are presented in Table 1. A total of 15,198 women of reproductive age were included in this study. The median (IQR) age at the time of the study was 28 (21–37) years, that at first sex 18 (16–21) years and that at first cohabitation 19 (17–22) years. The majority of the women were Christian (99.0%), and 95.3% had never used condoms. More than two-thirds (73.3%) of the participants lived in rural areas, and (72.4%) had good knowledge of HIV/AIDS transmission. About half (54.2%) were married, 53.3% were within the upper wealth index (Richer and Richest), and 41.8 % had completed at least primary education.

Table 2 shows the prevalence of HIV testing among the study participants. About two-thirds (68 %) of the women in this study reported never having been tested for HIV. Women who had ever been tested for HIV were markedly older at first sex, age at first cohabitation and current age than those who had not been tested for HIV. HIV testing and educational attainment were significantly associated. Higher percentages of women who had no formal education (21.9%) or had incomplete primary education (43.2%) had not been tested for HIV, compared to those with complete primary (12.2%), incomplete secondary (17.8%), complete secondary (2.9%) and higher education (2.0%). Interestingly, incomplete primary education was associated with a lower percentage than no education (43.2 vs. 21.9%). Undertaking an HIV test was also associated with religion, marital status, residence, wealth index, knowledge and condom use, with *p* < 0.001. Women with good knowledge of HIV transmission were more likely to undertake testing for HIV.

**Figure 2:**
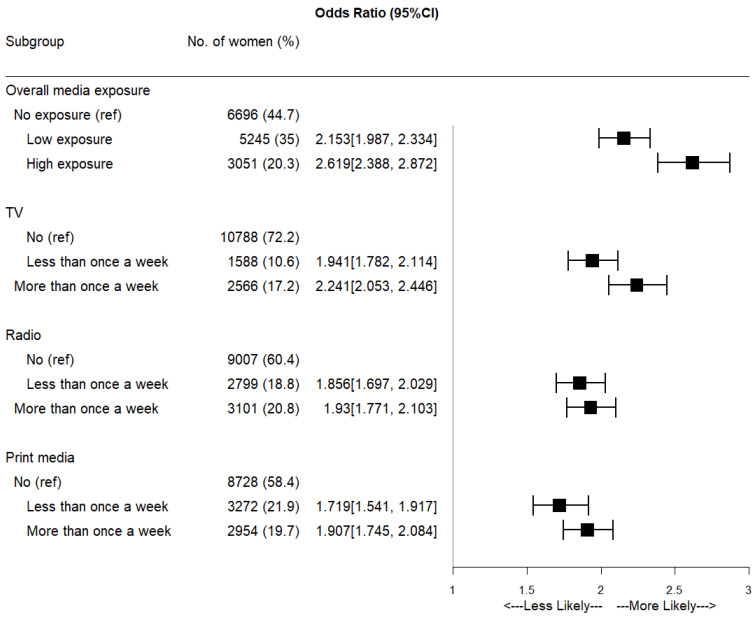
Forest plot showing the impacts of different media exposure on HIV testing.

Table 3 details the relationship between exposure to mass media and HIV testing. A higher proportion of women not exposed to mass media (55.3%) had not been tested for HIV compared to women with low exposure (29.0%) or high exposure (15.6%). Television is the medium least used among women in PNG (72.2%). Only about 17.2% reported watching television more than once a week, 20.8% reported listening to the radio more than once a week, and 19.8% stated they read print media at least once a week. Overall, we observed that women exposed to mass media were more likely to test for HIV compared to those with no exposure (69.0 vs. 31.0%). Figure 2 displays the relationship between individual exposure to media and HIV testing. Women with high exposure to TV, radio and print media were more likely to test for HIV. 

We also present the adjusted odds ratio of HIV testing while controlling for other covariates which may potentially confound the results. For example, after controlling for other covariates in the multivariable model, the effects of media exposure was still significantly high (Table 4). Women with increased media exposure were more likely to have had an HIV test (aOR = 1.53, 95% CI: 1.36, 1.72) compared to women with no media exposure. We also found a higher level of education was significantly associated with increased odds of HIV testing, and the odds were highest for women with more than secondary education (aOR = 3.38, 95% CI: 2.57, 4.46). Higher household wealth quintiles and condom use were associated with HIV testing, while marital status, knowledge of transmission and religion were not associated with HIV testing (Table 4).

## 4. Discussion

A significant outcome of this study is that exposure to mass media is associated with the willingness of women of reproductive age to be tested for HIV. In this study, we provide evidence that the level of education and knowledge about HIV transmission correlates with HIV testing. We found that women who used condom were 58% more likely to undergo HIV testing.

In the past few decades, the PNG government has initiated and implemented several policies to improve the rate of HIV testing; however, the majority of the population still remain untested, and this has prevented PNG from achieving the UNAIDS 90-90-90 target [22]. Our study shows a positive relationship between media exposure and HIV testing rate. We show that exposure to any form of media such as TV, radio and print increases the likelihood of HIV testing. This positive correlation is congruous with other findings in different countries [23,24]. Similarly, studies in Bangladesh and North India also showed a significant association between media exposure and HIV awareness [25,26]

With regard to behavioural risk factors among women in PNG, several studies support the involvement of behavioural risks factor in HIV testing among women of reproductive age [9] and previous knowledge of their HIV status [27]. We reported similar results; firstly, our analysis highlights the lack of condom use among the study participants. Furthermore, there was a significant association between condom use and HIV testing. The increase in condom non-users found in the present study might suggest an important target for future re-educational and interventional campaigns, which, if successful, would not only decrease the rate of HIV transmission per sexual contact but also increase the rate of HIV testing. Secondly, although the proportion of women in the study who had good knowledge of HIV transmission was high, this did not translate into a significant increase of odds of HIV testing. It would be reasonable to expect a higher level of HIV knowledge (transmission) to be associated with a higher level of HIV testing, as it stands to reason that the more knowledge of a particular disease a person has, the more aware they are of the importance, availability and processes of testing for that disease. A likely explanation for this finding could be that “knowledge” was assessed based on three Yes or No questions, and this mode of assessment might introduce the possibility of guessing among the participant, so that the results obtained might not be the precise representation of the actual knowledge among the participants. Another possibility is that most of the women lived in rural areas where it might be difficult to access or receive mass media such as radio signals, TV or publications, and this might contribute to their little or no education.

Higher educational attainment confers a greater probability for higher knowledge of transmission [28,29]. As expected, our study showed a strong association between a higher education level and HIV testing. This is consistent with a Zambian study, which showed a strong association between educational attainment and HIV testing among women of childbearing age [28]. The perception of risk related to safe sex might have contributed to the strong association of condom use with HIV testing observed in our study, though we observed no association of knowledge of HIV with HIV testing behaviour even when the variables were adjusted. This might suggest that the media may have a greater impact by adjusting societal norms and reducing barriers to testing rather than by educating people about HIV transmission [30]. This finding was also similar to our observation in the present study.

### Limitation and Strength

The present study is based on the 2019 PNG DHS data, and as such, its main strengths are akin to those of Demographic Health Surveys in general. DHS surveys produce generalisable data through standardisation of the collection methods and uniformity of the procedures, increasing their internal validity over repeated cycles in the same region, as well as their external validity on a global level. They collect a wide breadth of data, are economical and incorporate extensive quality controls and monitoring to detect and rectify errors at an early stage. Our study was limited to statistical associations based on the cross-sectional nature of the DHS data. HIV testing is a self-reported measure and thus could be subject to error, whilst HIV status and HIV testing may be underreported based on the sensitivity and stigma that exists in PNG. Other self-reported variables, such as media exposure and condom use, may also be subject to recall bias. The measure of media exposure was dichotomous, combining TV, radio and print media. The finer details of the effect of different levels of exposure to different types of media are unknown. Further, the current analysis did not consider the content of the media to which the participants were exposed and did not include social media, an ever-growing form of media consumption, as a source. It will be interesting to investigate the impact of different media modes on HIV testing, awareness and knowledge of HIV transmission among different genders and if there is a sex-specific effect on the behavioural risk for HIV testing.

## 5. Conclusions

HIV testing and counselling represents the cornerstone of HIV prevention and treatment and is considered one of the most cost-effective health promotion tools to reduce HIV transmission [19]. Mass media could be an effective and convenient tool for improving knowledge of HIV transmission and HIV testing. In the present study, we provide strong evidence that mass media exposure increases HIV testing among women aged 15–49 years in PNG. This indicates that media exposure and education attainment are effective tools that could foster HIV testing among women of reproductive age. Further studies should focus on the impact of media exposure on HIV testing in the male population and the potential role of social media and evolving media access patterns by gender.

## Figures and Tables

**Table 1:** Socio-demographics characteristics of the study population.

Variables		
Age at first sex, median (IQR) ^a^	18 (16–21)	
Age at first cohabitation, median (IQR) ^b^	19 (17–22)	
Women’s current age, median (IQR)	28 (21–37)	
	*n*	%
Educational attainment		
None	2815	18.8
Incomplete primary	5627	39.5
Complete primary	1870	12.5
Incomplete secondary	3249	21.7
Complete secondary	569	3.8
Higher	575	3.8
Religion ^c^		
Christian	14,737	99.0
Non-Christian	66	0.4
No religion	85	0.6
Marital status		
Never married	4138	45.8
Married	8134	54.2
Living with partner	1645	11.0
Widowed	230	1.5
Divorced/Separated	858	5.7
Residence		
Urban	3999	26.7
Rural	11,006	73.3
Wealth index		
Poorest	2108	14
Poorer	2277	15.2
Middle	2623	17.5
Richer	3667	24.4
Richest	4330	28.9
Knowledge (Transmission) ^d^		
None (0)	2613	17.3
Low (1–4)	3010	19.8
Good (5–8)	9527	62.9
Condom use ^e^		
No	8900	95.3
Yes	442	4.7

^a^ Missing 1.0%, ^b^ Missing 27.4%, ^c^ Missing 0.1%, ^d^ Missing 0.3%, ^e^ Missing 37.6%.

**Table 2:** Distribution of HIV testing among the study participants.

Variables	HIV Testing				*p*-Value
	Yes, *n* (%)		No, *n* (%)		
	4728 (32%)		10,277 (68%)		
Age at first sex, median (IQR)	19 (17–20)		18 (16–21)		<0.001 ^a^
Age at first cohabitation, median (IQR)	20 (17–22)		19 (17–22)		<0.001 ^a^
Women’s current age, median (IQR)	30 (24–36)		27 (19–37)		<0.001 ^a^
Educational attainment					
None	566	(12.0)	2247	(21.9)	<0.001 ^b^
Incomplete primary	1488	(31.5)	4439	(43.2)	
Complete primary	618	(13.1)	1254	(12.2)	
Incomplete secondary	1421	(30.1)	1828	(17.8)	
Complete secondary	267	(5.6)	302	(2.9)	
Higher	368	(7.8)	207	(2.0)	
Religion					
Christian	4587	(99.3)	10,150	(98.9)	<0.001 ^b^
Non-Christian	14	(0.3)	52	(0.5)	
No religion	19	(0.4)	66	(0.6)	
Marital status					
Never married	430	(9.1)	3708	(36.0)	<0.001 ^b^
Married	3242	(68.6)	4892	(47.5)	
Living with partner	608	(12.9)	1037	(10.1)	
Widowed	65	(1.4)	184	(1.8)	
Divorced/Separated	383	(8.1)	475	(4.6)	
Residence					
Urban	1765	(37.3)	2234	(21.7)	<0.001 ^b^
Rural	2963	(62.7)	8043	(78.3)	
Wealth index					
Poorest	357	(7.6)	1751	(17.0)	<0.001 ^c^
Poorer	497	(10.5)	1780	(17.3)	
Middle	705	(14.9)	1918	(18.7)	
Richer	1285	(27.2)	2382	(23.2)	
Richest	1884	(39.8)	2446	(23.8)	
Knowledge (Transmission)					
None (0)	0	(0)	2613	(25.6)	<0.001 ^c^
Low (1–4)	836	(17.7)	2126	(20.8)	
Good (5–8)	3892	(82.3)	5490	(53.7)	
Condom use					
No	3441	(93.8)	5459	(96.2)	<0.001 ^b^
Yes	227	(6.2)	215	(3.8)	

^a^ Mann–Whitney U test; ^b^ Chi-square test for independence; ^c^ Chi-square test for trend.

**Table 3:** Relationship between media exposure and HIV testing in the study participants.

Variables	HIV Testing				*p*-Value ^†^
	Yes, *n* (%)		No, *n* (%)		
	4721 (31.5%)		10,271 (68.5%)		
Media exposure ^‡^^,a^					
No exposure	1462	(31.0)	5234	(50.9)	<0.001
Low exposure	1970	(41.7)	3275	(31.9)	
High exposure	1289	(27.3)	1762	(17.2)	
TV					
No	2991	(63.6)	7797	(76.2)	<0.001
Less than once a week	631	(13.3)	957	(9.3)	
More than once a week	1084	(22.9)	1482	(14.5)	
Radio					
No	2335	(49.8)	6672	(65.3)	<0.001
Less than once a week	1102	(23.5)	1697	(16.6)	
More than once a week	1250	(26.7)	1851	(18.1)	
Print media					
No	2173	(46.1)	6555	(64.0)	<0.001
Less than once a week	1281	(27.2)	1991	(19.4)	
More than once a week	1259	(26.7)	1695	(16.6)	

^†^*p*-value based on Chi-square for independence. ^‡^ Media exposure is the sum of TV, radio and print media, ^a^ Missing 0.1%.

**Table 4:** Association between exposure to media and HIV testing and other covariates.

	Unadjusted OR (95% CI)	Adjusted OR (95% CI)
Media exposure		
No exposure	ref	
Low exposure	2.15 (1.98, 2.33) ***	1.63 (1.39, 1.90) ***
High exposure	2.61 (2.38, 2.86) ***	1.53 (1.36, 1.72) ***
Age		
Age at first sex	1.02 (1.01, 1.03) ***	1.00 (0.98, 1.01)
Age at first cohabitation	1.02 (1.01, 1.03) ***	1.01 (1.00, 1.03)
Women’s current age	1.02 (1.02, 1.03) ***	0.96 (0.96, 0.97) ***
Educational attainment		
None	ref	
Incomplete primary	1.33 (1.19, 1.48) ***	1.22 (1.06, 1.40) **
Complete primary	1.94 (1.70, 2.22) ***	1.56 (1.30, 1.87) ***
Incomplete secondary	3.08 (2.74, 3.45) ***	2.18 (1.85, 2.58) ***
Complete secondary	3.50 (2.90, 4.22) ***	2.33 (1.77, 3.09) ***
Higher	7.03 (5.80, 8.55) ***	3.38 (2.57, 4.46) ***
Religion		
Christian	ref	
Non-Christian	0.58 (0.31, 1.02)	0.59 (0.27, 1.21)
No religion	0.62 (0.36, 1.02)	0.67 (0.33, 1.30)
Marital status		
Never married	ref	
Married	5.71 (5.13, 6.38) ***	0.89 (0.78, 1.01)
Living with partner	5.06 (4.39, 5.83) ***	1.31 (0.63, 2.66)
Widowed	3.40 (2.49, 4.58) ***	1.01 (0.76, 1.33)
Divorced/Separated	6.95 (5.88, 8.22) ***	
Residence		
Urban	ref	
Rural	0.47 (0.43, 0.50) ***	0.72 (0.63, 0.82) ***
Wealth index		
Poorest	ref	
Poorer	1.37 (1.18, 1.59) ***	1.28 (1.07, 1.54) ***
Middle	1.80 (1.56, 2.08) ***	1.59 (1.33, 1.89) *
Richer	2.65 (2.32, 3.02) ***	2.23 (1.89, 2.65) ***
Richest	3.78 (3.33, 4.30) ***	2.63 (2.17, 3.19) ***
Knowledge (Transmission)		
None (0)	ref	
Low (1–4)	1.23 (0.97, 1.55)	1.23 (0.91, 1.68)
Good (5–8)	0.91 (0.73, 1.14)	0.88 (0.66, 1.19)
Condom use		
No	ref	
Yes	1.68 (1.38, 2.03) ***	1.58 (1.26, 1.99) ***

Ref: Reference category, * *p* < 0.05; ** *p* < 0.01; *** *p* < 0.001.

## Data Availability

This study was based on publicly available data sets from the DHS Program (www.dhsprogram.com).
